# The effects of bilingualism on stuttering during late childhood

**DOI:** 10.1136/adc.2007.134114

**Published:** 2008-09-09

**Authors:** P Howell, S Davis, R Williams

**Affiliations:** 1 Division of Psychology and Language Sciences, University College London, London, UK; 2 Department of Language and Communication Science, City University, London, UK

## Abstract

**Objectives:**

To examine stuttering by children speaking an alternative language exclusively (LE) or with English (BIL) and to study onset of stuttering, school performance and recovery rate relative to monolingual speakers who stutter (MONO).

**Design:**

Clinical referral sample with supplementary data obtained from speech recordings and interviews.

**Setting:**

South-East England, 1999–2007.

**Participants:**

Children aged 8–12 plus who stuttered (monolingual and bilingual) and fluent bilingual controls (FB).

**Main outcome measures:**

Participants’ stuttering history, SATS scores, measures of recovery or persistence of stuttering.

**Results:**

69 (21.8%) of 317 children were bilingual. Of 38 children who used a language other than English at home, 36 (94.7%) stuttered in both languages. Fewer LE (15/38, 39.5%) than BIL (23/38, 60.5%) children stuttered at first referral to clinic, but more children in the fluent control sample were LE (28/38, 73.7%) than BIL (10/38, 26.3%). The association between stuttering and bilingual group (LE/BIL) was significant by χ^2^ test; BIL speakers have more chance of stuttering than LE speakers. Age at stuttering onset and male/female ratio for LE, BIL and MONO speakers were similar (4 years 9 months, 4 years 10 months and 4 years 3 months, and 4.1:1, 4.75:1 and 4.43:1, respectively). Educational achievement was not affected by bilingualism relative to the MONO and FB groups. The recovery rate for the LE and MONO controls together (55%) was significantly higher by χ^2^ test than for the BIL group (25%).

**Conclusions:**

BIL children had an increased risk of stuttering and a lower chance of recovery from stuttering than LE and MONO speakers.

Bilingualism has been regarded as a risk factor for stuttering.^1^ ^2^ However, there is little information about how a second language affects the chances of stuttering onset and of recovery. Consequently, a study was conducted on all referrals for stuttering for children first seen when they were between 8 and 10 years of age who attended clinics that specialised in the diagnosis and treatment of stuttering. Children who used a second language in the pre-school years either (a) exclusively (these learned English at school, termed LE) or (b) along with English in the home (bilingual from birth, termed BIL) were selected. The majority of the children were seen again when they were aged 12 plus. Prevalence rates of stuttering for LE and BIL children as compared with all referrals were examined to determine if a disproportionate number of speakers of a language other than English is referred to stuttering clinics. Checks were made to verify that the LE and BIL children were stuttering by comparing age at stuttering onset and gender ratio for these speaker groups against monolingual speakers from a referral sample who stuttered (MONO). Early school performance of LE and BIL children who stuttered was compared with that of a MONO group and bilingual children who were fluent (the fluent bilingual group, termed FB). The patterns of onset and recovery in the LE, BIL and MONO groups were compared.

## METHODS

### Participants and sub-groups

A total of 317 children who stuttered participated. They all (a) started school in the UK at age 4 or 5, (b) first presented at a clinic when aged between 8 and 10 years and (c) lived in the greater London area. Stuttering was confirmed by a specialist speech-language therapist at the clinic. Reported onset of stuttering usually occurs before age 6. The attendance at clinic 2 or more years later is partly due to the time needed to process children in the health system; these are secondary referrals to the specialist clinic and devolved budgets to local area health authorities can cause delay. Recordings were taken to estimate the percentage of stuttered syllables and to allow the stuttering severity instrument measure SSI-3^3^ to be applied (see below for details of how SSI-3 was calculated).

When each child was seen initially, all 317 caregivers were asked whether they used a language other than English exclusively or sometimes in the home. In cases where use of a second language other than English was reported, the caregivers had a further interview. When the caregiver did not speak English, help with translation was obtained from a close friend who spoke the same language as well as English. The interview obtained background details about the child, the family and information about languages used in the home. Details were collected about the biological relationship of the caregiver to the child (all were parents of the child they cared for). Data on gender and age at onset of stuttering were obtained. The child’s Standard Attainment Test educational scores (SATS) at ages 7 and 11 (key stages one and two) were obtained for English, mathematics and science. It was also established whether the caregiver spoke English to the child from birth up until the child went to school, and whether care was given by this person exclusively or some of the time up to age 5 years. The caregivers indicated whether the child spoke English when they entered school. Information was obtained about where the child was born and, in cases where the child was an immigrant, the age at which they had entered the UK and their language use in their former country. This language information was used to classify children as LE or BIL.

There were 69 cases where at least one language other than English was spoken in the home. Caregivers of 38 of those 69 children reported that they primarily or exclusively used a language other than English in the home and had done so since the birth of their child. This criterion excluded people who opted to speak a language other than English in the home for their child’s educational/social advancement. All 38 families have continued to participate. Nine of the group of 38 have not yet reached 12 years, leaving 29 who were categorised as recovered from stuttering or not.

The 38 selected children were divided into LE and BIL groups. The LE children may be regarded as not being bilingual until the age of school onset. The LE group consisted of 15 children (39.5% of the 38 children) who did not speak English when they started school. During this period, English was not used by the caregivers, English-language media were not accessed in the home and if there were siblings, they did not speak English up to the age of school entry.

Twenty three (60.5%) of the children spoke English in addition to the primary language spoken in the home (BIL group). They were all exposed to English in the home from birth.

A group of monolingual English speaking children who stuttered (MONO) was selected in order to assess the impact stuttering has on epidemiology and education and to determine how the stuttering severity of the MONO group compared with that of the experimental groups. The MONO children were selected at random from the entire sample of such children referred to the clinic, subject to the restrictions that the group had to match the LE and BIL groups for age and gender. They had similar socio-economic backgrounds and attended similar schools to the LE and BIL children.

When children in all groups passed 12 years of age, they and their families were interviewed about their stuttering and school record, and the children were recorded so that a stuttering severity measure could be obtained (to confirm whether or not their stuttering had continued).

A second control group of fluent children who used a language other than English in their pre-school years was recruited (fluent bilingual, termed FB). These children were age-matched to those children who stuttered at second attendance, so that SATS scores could be compared. The children were recruited from schools in the same catchment areas as the clinics and they had similar educational and socio-economic backgrounds as the LE and BIL children, and reported no history of speech/hearing problems. They were divided into LE and BIL groups using the same criteria as above. Data from the LE and BIL groups of FB children were used to compare educational attainment levels with those obtained for LE and BIL children who stuttered.

### Classification as persistent/recovered

Stuttering was reassessed when the child was seen at age 12 plus by (a) the child, (b) the caregiver and (c) a researcher (the same for all children). All the scales used in these assessments have been normed (a statistical concept in psychometrics) (see supplementary data available online). The child questionnaire and the researcher report forms are given as supplementary material (the parent questionnaire was the same as the child questionnaire except that statements were changed from the first to the third person). For the child and caregiver, seven of the 15 questions on Boberg and Kully’s questionnaire^4^ were employed, and a further question was constructed which combined three more of their questions. For each of the eight questions, the child or caregiver indicated the extent of agreement on a five-point scale. Each question was scored 1–5, where 1 represented fluent behaviour and 5 dysfluent behaviour. The scores across all questions were summed, the maximum score being 40. Scores lower than 21 were considered “recovered” and scores greater than 21 were considered “persistent”. The scores on these questionnaires were correlated with SSI-3 scores as a validation criterion (part of the norming procedure described in the supplementary data). This showed that these cut-offs divided stutterers at the low end of the moderate scale (stuttering could not be moderate to have recovered and had to be at least moderate to be designated persistent).

The researcher visited each child’s home and recorded an interview that lasted approximately 90 min. During this visit, the researcher talked with a caregiver and the child about the speech problem and their experience in the clinic. He also sought their views about communication style and self-confidence in a range of typical environments. These included home and social gatherings with adults and children in and out of school. Performance and experience in school were assessed in terms of inter-personal relationships with staff and other children (including bullying). General health issues were also examined, including frequent absence from school and childhood illnesses. The researcher subsequently assessed speech fluency, social-conversational skills, and whether the child had a positive self-image/confidence about speech, using the recordings and notes taken at the home visit. Each of the three assessments was scored on a scale of 0 (good) to 3 (poor). The scores for the three factors were summed to give one score of between 0 and 9. A score of 5 or above indicated still stuttering.

To be designated as persistent, the caregiver, child and researcher all had to rate the child as still stuttering. To be designated as recovered, the caregiver, child and researcher all had to designate the child as not stuttering. All cases were unambiguously classified on all three criteria.^5^^–^8 All participants have been followed up for a minimum of 12 months and substantially longer in some cases (the mean length of follow-up for all speakers is 31.5 months with an SD of 24 months). The recovered participants showed no relapse and none of the participants designated as “persistent” recovered during this period.

The percentage of syllables stuttered out of the total syllables spoken in a 2 min recording of spontaneous speech made on the second occasion when the child was aged 12 plus was used to further check persistence/recovery. All recovered stutterers had fewer than 4% stuttered syllables, whereas all persistent stutterers had more than 4% stuttered syllables. Yairi and Ambrose reported that 3% stuttered syllables distinguished most speakers who stutter from fluent controls.^9^ From this, the recovered stutterers can be considered to be close to fluent whereas the persistent stutterers cannot.

### Stuttering severity assessment (SSI-3)

The SSI-3, a standard measure of stuttering severity, was administered when the child was first seen and again at age 12 plus.^3^ The assessments were always conducted on samples spoken in English, including samples from two children who did not stutter in English but did in their first language. This was because SSI-3 is not available for those children’s first languages and no norms exist. For SSI-3, a monologue, a dialogue and a text, all of which contained at least 200 syllables, were recorded using a Sennheiser K6 microphone and a Sony DAT recorder. Associated physical concomitants such as tics and twitches were noted. SSI-3 scores were obtained by qualified personnel.

### Statistical analysis

SPSS 11.0 (release 11.5.0; SPSS, Chicago, IL) was used for the descriptive analysis and for the non-parametric χ^2^ tests. For parametric measures either independent t tests (SATS performance categories) or ANOVA (age at onset, SATS absolute scores and SSI-3 scores) were used for assessing differences between groups. For the parametric measures, 95% confidence intervals were calculated and p<0.05 was considered to be significant.

### Ethics approval

Ethics approval was obtained from the UCL Committee on the Ethics of Non-NHS Human Research (project ref 0754/003).

## RESULTS

### Prevalence of LE and BIL in a sample of speakers who stutter

The prevalence of a second language in the sample was 69/317 or 21.8%, which compared to 28.4% of bilingual children in general reported by the London Education Authority. A goodness of fit between the obtained data and the estimates using 28.4% as the expected value for bilingualism was not significant (χ^2^ = 3.702, df = 1, p = 0.054). Thus, there does not appear to be a difference between the number of children who used a minority language in this sample and in London schools in general.

Two (5.3%) of the 38 LE and BIL children stuttered in only one language. They were both BIL children who did not stutter in English. This is in agreement with reports that show that for bilingual speakers who stutter, it is usual to stutter in one language only.^10^ ^11^

The number of LE children who stuttered (15) was lower than the number of BIL children who stuttered (23). However, when FB children were divided into LE and BIL groups using the same criteria, the reverse was true, with 28 LE and 10 BIL children. The association between LE and BIL and fluency group (children who stuttered/children who were fluent) was significant (χ^2^ = 9.051, df = 1, p = 0.003). Thus, a relatively high proportion of BIL children stuttered compared to those who were fluent.

### Individual profiles and family structure of LE and BIL children who stuttered compared to monolingual controls who stuttered

The groups of children who stuttered were compared on other characteristics to determine whether there were any differences between the LE, BIL and MONO children. Stuttering affects more males than females.^8^ ^9^ This was true of all three groups of children who stutter, with male/female ratios of 4:1, 4.75:1 and 4.43:1 for LE, BIL and MONO groups, respectively. A χ^2^ test showed that there was no association between these groups and gender, indicating that all three groups had the same gender imbalance towards males.

Stuttering usually starts early in development but some time after language onset.^8^ ^9^ The mean age at onset for the LE, BIL and MONO groups was 4 years 9 months, 4 years 10 months and 4 years 3 months, respectively. Again this corresponds with estimates obtained on other children in this age range,^8^ although children younger than those examined here report earlier onset.^9^ ^12^ ^13^ A between-groups ANOVA showed that age at onset of stuttering did not differ between the three groups. When onset and gender were compared, LE and BIL children were similar to MONO speakers who stutter.

**What is already known on this topic**

- Popular conception and early data suggest an increased risk of stuttering for bilingual speakers, although the data are sparse and this claim has been questioned recently.
- The Lidcombe treatment programme has been reported to be an effective treatment for young bilingual children who stutter.

**What this study adds**

- Children who are bilingual usually stutter in both their languages (rather than just one). If a minority language alone is used in the home up to age 5, the chance of starting to stutter is lower and the recovery rate is higher than for children who acquire English as well as a minority language during this period.
- Learning English concurrently with or after a minority language does not affect educational attainment at key stages one (age 7) and two (age 11).

### Early school performance of LE and BIL children relative to MONO and FB controls

The caregivers reported SATS scores for English, mathematics and science for the children at key stage one (age 7) and key stage two (age 11). By the age of 7 the children had not yet attended clinic but they had had a minimum of 2 years’ schooling.

SATS performance scores are published for each key stage and are classified as exceptional, beyond expectation, at the level expected and below expectation for the particular age group. Children were assigned to one of these categories separately for each subject examined (English, mathematics and language). Although performance levels would be expected to be lower for key stage one than for key stage two, the distributions can, nevertheless, still be compared. This was done for the LE, BIL, MONO and FB LE and FB BIL groups. The distribution of performance levels of the children across these groups was not significantly different by χ^2^ test (there was no association between SATS performance category across the five speaker groups, p = 0.865).

Separate ANOVAs were conducted for English, mathematics and science on absolute scores. For each of these analyses there was one between-group factor (speaker group with five levels: LE who stuttered, BIL who stuttered, MONO who stuttered, FB who were LE, FB who were BIL) and one within-groups factor (assessment stage with two levels: key stage one, key stage two). For all three assessment types there was a significant improvement across assessment stages (English, F(1,44) = 160.762, p<0.001; mathematics, F(1,44) = 102.271, p<0.001; science, F(1,44) = 129.175, p<0.001) but no effect of speaker group. The interaction between speaker group and assessment stage was marginally significant for mathematics (F(4,44) = 2.647, p = 0.046). This arose because the FB who were LE scored higher in mathematics at key stage one but fell back to the same level as the other speaker groups at key stage two.

### Severity assessment and recovery rates of LE and BIL children compared to MONO controls

**Figure 1:**
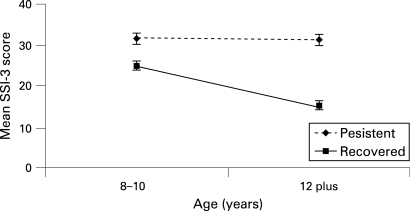
The SSI-3 scores of the recovered and persistent groups over these two ages.

SSI-3 severity estimates^3^ were examined at first attendance and at age 12 plus. The children were first divided into those who recovered and those who persisted in stuttering at age 12 plus, as severity measures were expected to differ across ages for the two types. The SSI-3 scores were examined in a three-way ANOVA with two between-groups factors (factor 1, speaker groups, has three levels: LE, BIL and MONO; factor 2, recovery type, has two levels: persistent or recovered) and one within-group factor (age with two levels: 8–10 and 12 plus). There was a significant decrease over ages: F(1,44) = 29.119, p<0.001. There was also a significant main effect of recovery type, F(1,44) = 40.639, p<0.001, but no significant main effect of speaker group, F(2,44) = 0.186, p = 0.836. There was also a significant interaction between age and recovery type, F(1,44) = 51.785, p<0.001. Figure 1 shows this interaction on SSI-3 scores for persistent and recovered stutterers at the two ages. The SSI-3 scores of the persistent groups were about the same across the two ages (scores around 30 points).The SSI-3 scores of the recovered group decreased from about 25 points at the first test age to around 15 at the second test age.

Stuttering tends to persist in BIL speakers and there is a higher risk of such children starting to stutter. The LE and MONO groups were collapsed together because the numbers in the LE group were low when these speakers were divided into persistent and recovered cases, and the patterns in the collapsed groups were similar. There was a significant association between speaker group (BIL and LE-MONO) and recovery outcome (persistent/recovered): χ^2^ = 4.381, df = 1, p = 0.036. The BIL group had a 25% recovery rate, whereas the LE and MONO groups together had a 55% recovery rate. These and other statistics are summarised in table 1.

**Table 1:** Summary of statistical tests performed

(A) Comparisons between LE and BIL speakers					
Stutterers	M/F ratio	% Stutter in one/both languages	Age at stuttering onset	SATS	SSI-3
LE, n = 15	4:1	0%	4 years 9 months	Same as all peers	Same as other stutterers
BIL, n = 23	4.75:1	5.3%	4 years 10 months	Same as all peers	Same as other stutterers
	NS	–	NS	NS	NS

(B) Comparison between all bilingual stutterers and bilingual non-stutterers					
	Prevalence of a second language in respective sample				
Bilingual stutterers	21.8%				
Bilingual non-stutterers	28.4%				
	NS				

(C) Comparisons between stuttering and non-stuttering groups					
	Incidence of LE and BIL	Recovery rate of LE and BIL			
Stuttering sample	Fewer LE than BIL	Recovery rate of LE better than that of BIL			
Non-stuttering sample	More LE than BIL	–			
	Significant	Significant			

BIL, bilingual from birth; LE, learned English at school; M/F, male/female; NS, not significant; SATS, Standard Attainment Test educational score; SSI-3, Stuttering Severity Instrument, Third Edition.

## DISCUSSION

The incidence of bilingualism in the clinical sample was 21.8%. This was roughly comparable with the reported incidence of bilingualism in the same geographical area (28.4% of pupils in the area covered by London Educational Authority are bilingual). Stuttering in one language by bilingual children is rare (only 5.3% of the sample of 38 bilingual children stuttered in just one of their languages).^10^ The similar gender ratios (all around 4:1 for the LE, BIL and MONO groups) and the similar reported age at stuttering onset (around 4½ years for all groups) supported the view that all groups stuttered.

The BIL group was particularly prone to starting to stutter. Thus, at the age at which the children first attended clinic, there were more BIL than LE children, whereas the reverse was the case in a control fluent sample. The statistics supported the view that there was a higher chance of BIL children starting to stutter as the association between stuttering/not stuttering and BIL/LE was significant. This showed that BIL and LE groups were distributed differently in the stuttering, compared to the non-stuttering, sample (60.5% of the stuttering group were BIL, whereas only 26.3% of the non-stuttering group were BIL). The BIL group also had a lower chance of recovery. The recovery rate at 12 plus was higher for the LE and MONO groups combined than for the BIL group. The statistics supported the position that BIL children had a lower chance of recovery as there was a significant association between the BIL versus the LE-MONO group and recovery outcome. Inspection of the data revealed that only 25% of the BIL group recovered whereas 55% of the LE-MONO group recovered. Together, these findings suggest that if a child uses a language other than English in the home, deferring the time when they learn English reduces the chance of starting to stutter and aids the chances of recovery later in childhood. A final factor of note is that school performance was not affected with respect to whether the child stuttered or not.
